# Insights into protein sequencing with an *α*-Hemolysin nanopore by atomistic simulations

**DOI:** 10.1038/s41598-019-42867-7

**Published:** 2019-04-23

**Authors:** Giovanni Di Muccio, Aldo Eugenio Rossini, Daniele Di Marino, Giuseppe Zollo, Mauro Chinappi

**Affiliations:** 1 Dipartimento di Ingegneria Industriale, Università di Roma Tor Vergata, Via del Politecnico 1, 00133 Roma, Italy; 2 Dipartimento di Scienze di Base e Applicate per l’Ingegneria, Università di Roma “La Sapienza”, Via A. Scarpa 14–16, 00161 Rome, Italy; 3 Faculty of Biomedical Sciences, Institute of Computational Science - Center for Computational Medicine in Cardiology Università della Svizzera Italiana (USI), Lugano, Switzerland; 4 Polytechnic University of Marche, Department of Life and Environmental Sciences, Via Brecce Bianche, 60131 Ancona, Italy

**Keywords:** Nanopores, Protein sequencing

## Abstract

Single molecule protein sequencing would represent a disruptive burst in proteomic research with important biomedical impacts. Due to their success in DNA sequencing, nanopore based devices have been recently proposed as possible tools for the sequencing of peptide chains. One of the open questions in nanopore protein sequencing concerns the ability of such devices to provide different signals for all the 20 standard amino acids. Here, using equilibrium all-atom molecular dynamics simulations, we estimated the pore clogging in *α*-Hemolysin nanopore associated to 20 different homopeptides, one for each standard amino acid. Our results show that pore clogging is affected by amino acid volume, hydrophobicity and net charge. The equilibrium estimations are also supported by non-equilibrium runs for calculating the current blockades for selected homopeptides. Finally, we discuss the possibility to modify the *α*-Hemolysin nanopore, cutting a portion of the barrel region close to the trans side, to reduce spurious signals and, hence, to enhance the sensitivity of the nanopore.

## Introduction

Nanopores have been demonstrated a great versatility in biosensing, as they can be employed to detect and analyze biological sample at single molecule level^1–14^. In nanopore sensing, the interaction of the molecule with the nanopore, e.g. its translocation through the pore, alters one or more properties of the system that can be recorded by appropriate instruments. The most commonly used approach is the so called resistive pulse, where the changes in the nanopore electric resistance induced by the molecule are associated to molecule properties. Another promising approach, based on tunneling effect, measures the alteration of the transverse current along the membrane plane^5,15–17^.

A potentially disrupting application of nanopore sensors is the single molecule protein sequencing. Compared to DNA sequencing, nanopore protein sequencing poses several challenges due to the large number of monomers to be distinguished (20 amino acids with respect to to 4 bases), the non-uniform charge of the polipeptidic chains (amino acid forming the protein can be neutral, positively or negatively charged) and the complex structure of proteins and peptides. The last two features make difficult even the mere capture of the protein by the nanopore. Indeed, since proteins can be both positively and negatively charged, electrophoresis is not usable to induce the capture unless specific charged tags are added to the protein terminals^18,19^. To overcome this difficulties, other approaches, such as dielectrophoretic trapping^20–24^ and electro-osmotic flow^6,7,25,26^ have been proposed. In addition, the complex interplay between unfolding, capture and translocation typically results in a non-homogeneous multistep co-translocational unfolding process^18,27–32^.

Once the molecule is captured, the fundamental requirements for a nanopore based sequencing devices are, in essence, two^11^. (i) *Signal-to-monomer matching*. Each signal has to be unambiguously associated to a specific monomer in the protein sequence. In sequencing strategies where the entire chain is sequentially imported inside the pore, this implies that the translocation speed needs to be controlled. Kennedy *et al*.^8^ showed that a homogeneous translocation can be achieved using sodium dodecyl sulfate (SDS), an anionic compound that denaturates the protein providing it with a negative charged shell. They found that the current trace associated to the protein translocation has a number of peaks close to the number of amino acids of the analyzed protein. On the computational side, the possibility to exploit the adhesion of the peptide chain on 2D materials to get a step-like translocation^33,34^ has been recently explored as a possible approach to control the protein transport through the pore. (ii) *Distinguishability*. The signal level associated to a single amino acid (AA) has to allow the unambiguous identification of the AA. Several experimental^8–10,20,35^ and computational^33,35,36^ works have shown that also very small changes in peptide composition can be potentially detected by nanopores. In this respect, systematic analysis of the capability of nanopores to distinguish among all the different residues are highly needed, a remarkable recent example being the work by Farimani *et al*.^36^ on the computational assessment of the peptide sequencing capability of a MoS_2_ pore.

In the present study, we focused on the distinguishability of different AA in *α*-Hemolysin (*α*HL), the most widely employed pore in nanopore sensing^2,18–20,35,37,38^. To this aim, as a preliminary case study, we analyzed the differences of homopeptide chains inserted in the *α*HL. First, we employed an extensive set of non-equilibrium all-atom MD simulations ($\simeq 8\,\mu s$ in total) to calculate the current levels associated to four different neutral homopeptides. Our results show that, as expected, large residues correspond to lower current values. Interestingly, we find that an equilibrium quantity derived from continuum quasi-1D argument and indicated as “pore clogging estimator” is linearly correlated to the measured current blockages. The estimation of relative conductance is a factor four less computational demanding with respect to non equilibrium runs allowing us to explore all the standard amino acids. Our results show that *α*HL pore clogging is affected not only by amino acid volume, but also by hydrophobicity and net charge. In particular, charged residues leave more room to electrolyte motion compared to uncharged one, hence, for similar residue volume, they give rise to a smaller clogging.

## Results and Discussion

### Ionic currents for selected homopeptides

**Figure 1:**
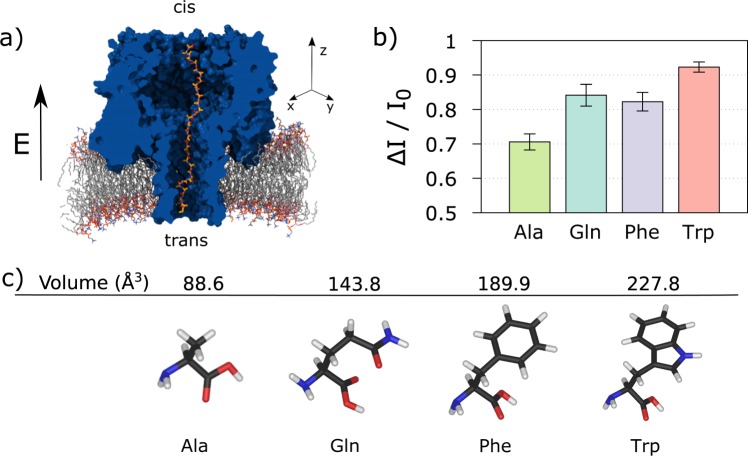
Ionic current measurements. (**a**) The system is constituted by an *α*- Hemolysin (*α*HL, blue) nanopore embedded into a lipid membrane (gray). A 35-residues homopeptide (orange chain) is imported into the nanopore with the central residue close to the pore constriction. The simulation box is filled up by 2M KCl electrolyte solution, that, for the sake of clarity, is not shown. A constant and homogeneous external electric field **E** = (0, 0, *E*_z_) parallel to the pore axis is applied. (**b**) Average current blockage Δ*I*/*I*_0_ = (*I*_0_ − *I*)/*I*_0_, with *I* the average current measured with the homopeptide inside the pore and *I*_0_ the empty pore value, for four different homopeptides, Ala, Phe, Gln, Trp. The data are obtained averaging the current blockades of five replicas for each homopeptide. Error bars are estimated by considering current blockades from independent replicas as independent measurements. (**c**) Molecular structure and Van der Waals volume of the four amino acids^45^.

We studied the ionic current for four different homopeptide chains clogging the *α*HL nanopore via all-atom molecular dynamics simulations. The four amino acids composing the homopeptides are alanine (Ala), phenylalanine (Phe), tryptophan (Trp) and glutamine (Gln) and, for each of them, we prepared five independent replicas. The system set-up is sketched in Fig. 1a. The *α*HL nanopore is embedded into a double-layer lipid membrane and immersed in a 2*M* KCl electrolyte solution, for a total of about 310 K atoms. After equilibration, the homopeptide is imported into the pore using steered molecular dynamics^39^. The frame with the central residue closer to the main pore constriction (Glu 111) is selected as starting configuration for the production runs. A constant and homogeneous external electric field *E* = (0, 0, *E*_z_) corresponding to a trans membrane voltage Δ*V* = 1 *V* is applied parallel to the pore axis. Each simulation was run for 240 ns and ionic current is estimated as the time average after discarding an initial transient of 64 ns, see Methods. The current blockage is defined as Δ*I*/*I*_0_ = (*I*_0_ − *I*)/*I*_0_, with *I* the average current measured with the homopeptide inside the pore and *I*_0_ the empty pore value.

Figure 1b shows the average current blockage Δ*I*/*I*_0_ for each homopeptide, which are obtained averaging the current blockade of 5 replicas for each homopeptide, while error bars are estimated by considering current blockades from independent replicas as independent measurements. Fig. S1a, instead, reports Δ*I*/*I*_0_ for each single replica. As expected, Δ*I*/*I*_0_ roughly reflects the steric hindrance of each amino acid, see Fig. 1c. Indeed, the lower blockage corresponds to Ala (VdW volume, *V*_A_ = 88.6 Å^3^)^40^ and the largest to Trp (*V*_W_ = 227.8 Å^3^) while Gln (*V*_Q_ = 143.9 Å^3^) and Phe (*V*_F_ = 189.9 Å^3^) blockages are in between the Ala and Trp values.

Interestingly, significant differences among replicas of the same homopeptide are found for Ala, Gln, and Phe, while, Trp replicas do not show any significant variability, see Section S1 and Fig. S1b of Supporting Information. This occurrence can be explained in term of the capability of smaller amino acids to explore a larger number of conformations inside the pore.

### Electrolyte occupancy

The above presented results indicate that the size of the side chain is correlated to the current blockage; the larger the side chain, the deeper the current drop. A similar results was find also for DNA and Mpsa (another biological pore used for sensing), by Bhattacharya *et al*.^41^ where it was shown that the number of water molecules displaced from the nanopore by the DNA determines the ionic current blockade, whereas the steric and conformational (base-stacking) properties of the DNA determine the amount of water displaced.

To better investigate the role of peptide conformation on the current drop, we formulated the following simple theoretical model. In a quasi-1D continuum description, the pore resistance is expressed as

1 $$R={\int }_{0}^{L}\frac{\rho (z)}{A(z)}\,dz,$$

where the *z*− axis coincides with the pore axis, the pore goes from *z* = 0 to *z* = *L*, *ρ*(*z*) is the electrolyte resistivity and *A*(*z*) is the area of the pore section available to the electrolyte. Access resistances are neglected.

**Figure 2:**
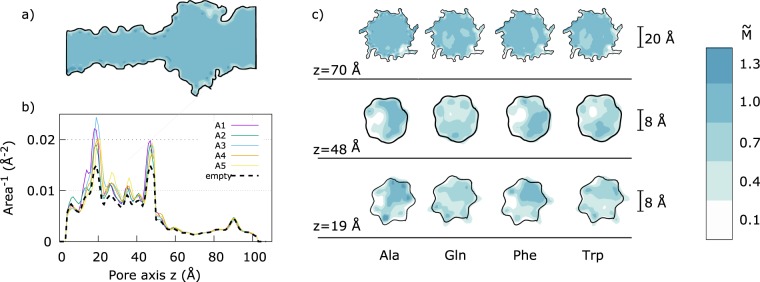
Accessible volume estimation. (**a**) The panel report a cut of the 3D averaged occupancy map ${\tilde{M}}_{{\rm{x}},{\rm{y}},{\rm{z}}}$ for the empty pore. Blue areas corresponds to region that are fully accessible by the electrolyte ${\tilde{M}}_{{\rm{x}},{\rm{y}},{\rm{z}}}=1$ while white ones do not contribute to the volume useful for the ions transport between the two side of the membrane, ${\tilde{M}}_{{\rm{x}},{\rm{y}},{\rm{z}}}=0$. (**b**) Inverse of the accessible area, *A*_z_, along the pore. The empty pore profile is plotted as dashed black line. The two peaks at $z\simeq 50$ Å and $\simeq 20$ Å correspond to the central *α*HL constriction and to the constriction close to the barrel entrance, respectively. The five solid lines refer to the five Ala replicas. (**c**) Slices of ${\tilde{M}}_{{\rm{x}},{\rm{y}},{\rm{z}}}$ normal to the pore *z*-axis passing through the two constrictions (*z* = 19 Å and *z* = 48 Å) and the vestibule (*z* = 70 Å) for the four homopeptides.

To estimate *A*(*z*) from our non-equilibrium runs, we divided the system in cubic cells of size Δ*x* = Δ*y* = Δ*z* = 1 Å, and, for each frame, we used the VMD Volmap plug-in^42^ to compute the occupancy map of the electrolyte, *m*_x,y,z_, where *x*, *y*, *z* indicate the cell, *m*_x,y,z_ = 1 if the cell is within a Van der Waals radius of at least one water or ion atoms and *m*_x,y,z_ = 0 elsewhere. Then, we averaged *m*_x,y,z_ over all frames and normalized it with the bulk value. The resulting averaged and normalized occupancy map is indicated with *M*_x,y,z_. As already discussed in Aksimentiev and Schulten^38^, “electrolyte pockets” are present close to constriction, see Fig. S2. The pockets do not contribute to the ion current. To filter out these pockets, we defined a trans → cis available channel as the pore region accessible to the electrolyte when moving from the barrel entrance towards the vestibule. This procedure excludes reentrant pockets directed towards the trans side, see Fig. S2 of Supporting Information. The same procedure is applied to get a cis → trans accessible pore, and the final occupancy map ${\tilde{M}}_{{\rm{x}},{\rm{y}},{\rm{z}}}$ is obtained as the intersection of the trans → cis and cis → trans accessible pores, see section S2 of Supporting Information for details. Figure 2c reports slices of ${\tilde{M}}_{{\rm{x}},{\rm{y}},{\rm{z}}}$ for the four homopeptides. The regions available for the electrolyte transport between the two sides of the membrane are indicated in blue.

The occupancy map ${\tilde{M}}_{{\rm{x}},{\rm{y}},{\rm{z}}}$ allows direct estimation of the pore section *A*(*z*) that can be calculated summing ${\tilde{M}}_{{\rm{x}},{\rm{y}},{\rm{z}}}$ on slices of width Δ*z* normal to the pore axis, in formula

2 $${A}_{z}=\sum _{{\rm{x}},{\rm{y}}}\,{\tilde{M}}_{{\rm{x}},{\rm{y}},{\rm{z}}}\,{\rm{\Delta }}x\,{\rm{\Delta }}y\mathrm{.}$$

Consequently, the resistance, Eq. (1), can be approximated as

3 $$\tilde{R}=\sum _{i=1}^{{N}_{{\rm{z}}}}\,\frac{\rho }{{A}_{{\rm{z}}}}{\rm{\Delta }}z,$$

where *i* = 1 and *i* = *N*_z_ correspond to the slice at pore trans and vestibule entrances, respectively, and we assumed that the resistivity *ρ* is constant along the pore. A similar quasi-1D model was recently applied in^43^.

Figure 2b reports the inverse of the available section profile, ${A}_{{\rm{z}}}^{-1}$ for the five Ala replicas (solid lines) and for the empty pore (black dashed line). In the vestibule region, *z* ∈ (60, 100) Å, the difference between the empty and the clogged *α* HL is negligible, indicating that, the contribution of the moiety of the homopeptide in the vestibule region to the pore resistance, Eq. (3), is almost unrelevant. More evident differences are present in the barrel region, *z* ∈ (5,50) Å, and, in particular in the main *α*HL constriction, $z\simeq 50$ Å. Interestingly, *A*(*z*)^−1^ has also a peak for $z\simeq 20$ Å. This is due to the non-polar Leu-135 residues that forms an isolated hydrophobic ring inside the barrel. Since the available section for the electrolyte passage is smaller in this region, we will indicate it as secondary barrel constriction. The hydrophobic nature of Leu-135 ring was shown to be relevant for DNA translocation through *α*HL^44^.

**Figure 3:**
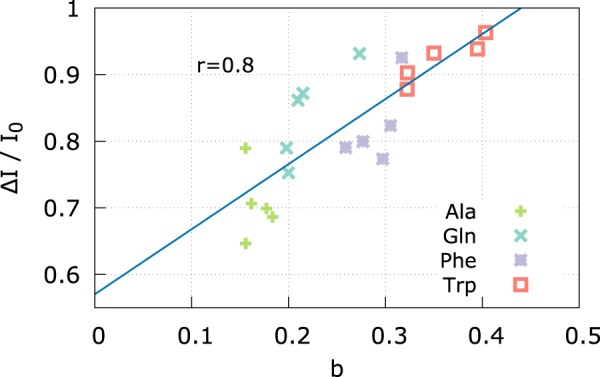
Pore clogging *b* Vs measured current blockage Δ*I*/*I*_0_. Linear regression curve is reported in dashed blue, Pearson correlation coefficient *r* = 0.8.

To quantify the correlation between the pore clogging and the ionic current, we defined the pore clogging estimator as

4 $$b=1-\frac{{\tilde{R}}_{0}}{\tilde{R}},$$

where ${\tilde{R}}_{0}$ refers to the empty pore. Equation (4) is inspired by the definition of the current blockage. Indeed, Δ*I*/*I*_0_ = 1 − *I*/*I*_0_, hence, using Ohm law, Δ*I*/*I*_0_ = 1 − *R*_0_/*R*. The above discussed model is based on several hypotheses that are violated by the actual *α* HL pore shape. In particular, the continuum assumption is not justified at nanoscale, moreover, the model implicitly assumes a smooth variation of *A*(*z*) along the pore axis. In addition, we considered a homogeneous electrolyte resistivity *ρ*. Nevertheless, although a strict quantitative agreement with the blockage Δ*I*/*I*_0_ and *b* is not expected, *b* results to be highly correlated with the measured Δ*I*/*I*_0_ (Pearson correlation coefficient *r* = 0.8), see Fig. 3.

### Pore clogging for all amino acids

Stimulated by the good correlation among the measured Δ*I*/*I*_0_ and the pore clogging estimator *b*, Eq. (4), we looked for a less computational demanding strategy to estimate *b*. We repeated the protocol described in the previous section using, as input data, 64 ns equilibrium runs (*E*_z_ = 0, only last 32 ns used for statistics) instead of the original 240 ns non-equilibrium trajectories. The resulting equilibrium pore clogging estimator is indicated as *b*^*eq*^. The result discussed in section S3 of the supporting information, show that, although the value of *b* slightly changes when using equilibrium or non equilibriums runs as input, the correlation is still very good for Ala, Gln and Trp while deviations are obtained for Phe that, at equilibrium, show a larger clogging compared to non equilibrium runs. Figure S3c reports the equilibrium clogging profile for Phe. It is apparent that for replica F1 the clogging in the main *α* HL constriction is much higher than for the other replicas. We argue that this single outlier is responsible of the high deviation from equilibrium and non equilibrium average clogging for Phe.

**Figure 4:**
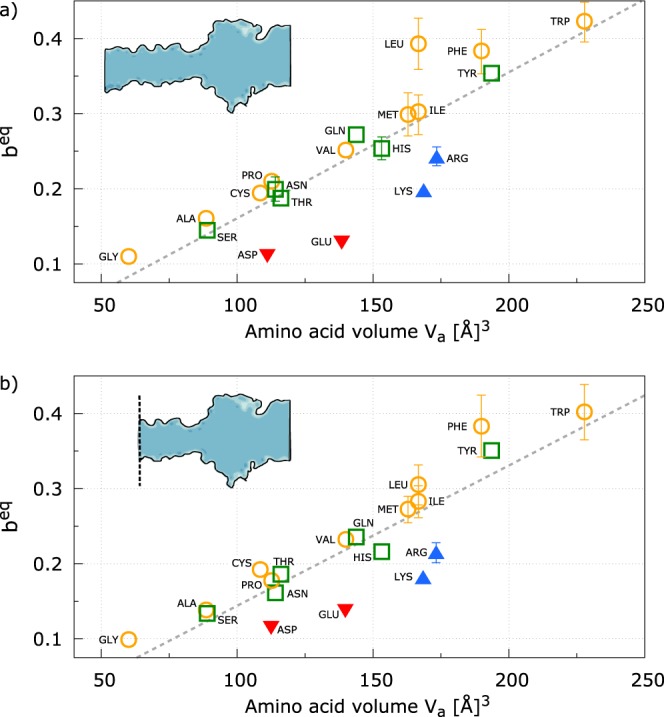
Pore clogging estimator *b*^*eq*^ for all residues Vs the amino acid volume *V*_a_. Yellow circles corresponds to hydrophobic residues, green squares to polar, blue up-triangles to positively charged residues and red down-triangles to negatively charged ones. The dashed line is the minimum square fit. Panel *a* reports the *b*^*eq*^ calculated on the entire pore while panel *b* refers to the *b*^*eq*^ calculated removing the last part of the barrel including the secondary constriction, see the sketch in the inset. Error bars are estimated by considering *b*^*eq*^ from independent replicas as independent measurements and they are reported only when larger than symbols.

The relatively small computational cost of the equilibrium simulations needed to estimate *b*^*eq*^ allows us to explore the blockage features of all the amino acids. For each homopeptide, we run five different replicas. Figure 4a shows the pore clogging estimator *b*^*eq*^ Vs the apparent amino acid volume *V*_a_ ^45^. A very good correlation is evident for all uncharged residues, while charged residues lie below the regression line. Indeed, charged residues leave more room to electrolyte solution compared to uncharged one. Similarly, although less evident, polar residues (green) show, on average, a lower *b*^*eq*^ than hydrophobic ones *b*^*eq*^, see section S4 of the Supplementary Material for a statistical analysis. This occurrence can be explained as a combination of two concomitant effects. First, hydrophobic, hydrophilic and charged residues affect the structure of the first shells of the electrolyte solution surrounding them in different ways. Indeed, concerning water molecules, hydrophilic and charged residues induce a more compact layering with respect to hydrophobic ones, see, e.g.^46^. Secondly, charged peptides are slightly more stretched with respect to uncharged residues (see, section S5), increasing the effective cross-section of the clogged pore available for electrolyte motion. In addition, we observed that charged homopeptides also induce an overall increases of the total number of ions inside the pore. Indeed, the ratio between ions and water molecules inside the narrow pore region (barrel plus constriction) is 0.061 ± 0.002 for charged residues and 0.035 ± 0.001 for uncharged ones. These values can be compared with the empty pore one, 0.041, indicating that, despite the confinement, charged residues are able to partially carry their counterion shells inside the narrowest regions of the pore. In summary, on average, for a similar amino acid volume, the pore clogging is minimum for charged homopeptides and it progressively increases moving to hydrophilic and hydrophobic residues. This evidence suggest that, although the main feature controlling the pore clogging is the volume of the amino acid, also charge and hydrophobicity play a role. For completeness, Fig. S4 reports the inverse area profiles for each charged residue and for the corresponding hydrophobic residue with a similar volume while the correlation of the amino acid accessible surface area *S*^47^ and pore clogging *b*^*eq*^ is reported in Fig. S5.

Concerning the uncharged residues, the more evident outlier in Fig. 4a is Leu. Indeed, although its volume is the same of its isomer Ile, *b*^*eq*^ is much larger. A close inspection to the inverse of the available section profile 1/*A*(*z*) indicates that this discrepancy is mainly due to clogging of the secondary barrel constriction, $z\simeq 20$ Å. In particular, we observed that in some replicas, the Leu-homopeptide forms a short *α*− helix in the portion that occupies the secondary constriction, see Fig. S6.

The effect of secondary barrel constriction can be, in principle, eliminated using a truncated *α*HL as the one reported in^48^ where it was shown that *α*HL pores are stable also when the large portion of the trans side of the barrel are deleted. We explored this possibility with our model calculating the summation in Eq. (3) only for the *α* HL region going from the residues Ile 136 to Asn 123, approximatively 20 Å from the native trans barrel entrance, to the vestibule. Figure 4b reports the corresponding *b*^*eq*^ Vs *V*_a_ plot where Leu lies close to the regression line.

It is worth noting that recent experiments indicate that *α*HL is able to distinguish among three-block peptides where the central neutral residues were Alanine and Triptophan^35^, or Isoleucine and Serine^49^. Moreover, very recently Piquet *et al*.^9^, showed that also Aerolysin nanopore is able to discriminate between two different ten-residue long homopeptides made by Arginine (R) and Lysine (K) and one heteropolymeric peptides, (K)_5_-(R)_5_. Taken together, the cited experimental results and our simulations suggest that biological pores can potentially been employed for protein sequencing although several challenging issues, such as the translocation control, need to be solved^11^.

## Conclusion

Nanopore based protein sequencing devices have two fundamental requirements: (i) the *signal-to-monomer matching*, which implies that the capture and the translocation speed needs to be controlled, and the *distinguishability* of the signals associated to the different amino acids^11^. In the present study, we focused on the distinguishability of different amino acids in *α*-Hemolysin. As a preliminary case, we studied homopeptides occupying the whole pore. We first performed an extensive set of non-equilibrium all-atom MD simulations to calculate the ion current blockade induced by four different homopeptides. Inspired by a quasi-1D model for pore conductance, we defined the pore clogging estimator and showed that it correlates with the observed current blockage from non-equilibrium runs. The estimation of relative conductance is a factor four less computational demanding than non-equilibrium runs allowing us to explore all the 20 standard amino acids. Our results show that amino acid volume is the main feature that rules the pore clogging and, consequently, the current blockage. In addition, our results indicate that also hydrophobicity plays a role. Indeed, for similar amino acid volumes, charged residues are associated to a smaller pore clogging than uncharged ones and slight, but significant, differences are observed also between hydrophobic and polar amino acids. Our results suggest that *α* HL is potentially able to discern among the different residues. For some set of residues with very similar volume, however, the pore clogging is very close and the expected current blockage signal as well. In these cases, long current recordings and signal post processing would be required to distinguish among them^11^.

Furthermore, our study provides a set of structural and chemical-physical information about nanopore protein sequencing that can pave the road to improve the distinguishability of the signal associated to a single amino acid. Our simulation protocol can be easily generalized to other pores or to systematically study the effect of modifications of *α*HL pore with the aim to propose mutations that can be *ad hoc* designed to amplify the signal differences among the 20 amino acids or to reduce the noise (as discussed, for instance, concerning the cut of the last part of the barrel)^48^. Indeed, this kind of membrane protein engineering is already routinely used with different biotechnological applications.

### System setup

All-atom Molecular Dynamics (MD) simulations were performed using the NAMD software^50^. The CHARMM36 force field^51^ was employed to model lipid, protein, and TIP3P water molecules^52^. NBFIX corrections were applied for ions^53^.

The membrane-*α*HL system has been assembled using a protocol similar to the one used in other works^38,54,55^. In brief, the system was built starting from the *α*HL crystal structure PDB_ID: 7AHL^37^ downloaded from the OPM database^56^. The POPC lipid membrane, the water molecules, and the ions for neutralizing the system were added using VMD^42^. Then, the system is minimized and a 60 ps NVT simulation (time step 0.2 fs) was run with external forces applied to the water molecules to avoid their penetration into the membrane and the pore. Lipid heads have been constrained to their initial position by means of harmonic springs (spring constant *k* = 1 *kcal*/(*mol*^2^)) acting on the phosphorus. A second equilibration run (1 ns NPT flexible cell, time step 1 $\tilde{{\rm{f}}}$s) was performed to compact the membrane. During this run, the lipid heads were unconstrained. The third, and last, equilibration step consists of a NPT constant area simulation (2 ns, time step 2 fs) where all the atoms are unconstrained and no external forces act on the water molecules. The resulting periodic box, after the equilibration, has the following size: *L*_x_ = 127.5 Å, *L*_y_ = 127.1 Å, and *L*_z_ = 180.0 Å, and the total number of atoms is ~290000. Initial configurations of peptides are generated by using the PEPFOLD server^57^ and then separately equilibrated in a triperiodic water box. Then, the two systems were merged, ions (2*M* KCl) were added using VMD, and a short NPT equilibration is performed (2 ns, constant area NPT) until *L*_z_ reaches a stationary value. The resulting box has dimensions *L*_x_ = 127.5 Å, *L*_y_ = 127.1 Å (i.e. the same as the original equilibrated *α*HL-membrane box) while ${L}_{{\rm{z}}}\simeq 186.2$ Å (slightly different values are get for each homopeptide) and the overall number of atom is ~310000.

### Peptide insertion

For each replica, a dedicated Steered Molecular Dynamics simulations was employed to bring the peptides at the pore’s lumen entrance (trans side) and, then, into the pore. In particular, the peptide N-terminus was placed at ~15 Å from the *α*HL’s trans entrance and then pulled inside the nanopore using a constant velocity Steered Molecular Dynamics (SMD) simulation at pulling speed *v*_SMD_ = 0.025 Å/ps.

The total SMD simulation time is *t*_SMD_ = 17 ns that corresponds to a motion of the pulled atom of Δ_SMD_ = 425 Å during which the peptide crosses the *α*HL two times. The initial configurations for the subsequent non equilibrium (**E** > 0) and equilibrium (**E** = 0) production runs for, respectively, ionic current and *b*^*eq*^ measurements (see next section) were chosen among the ones of the second passage. Since such SMD method can force the polymer to adopt highly stretched conformations^58^, we checked that the homopeptide relax toward equilibrium by computing the time evolution of gyration radius. The average relaxation time is $\simeq 10$ ns, see Section S6 and Fig. S7 of Supporting Information for details, hence, in the production runs, we discarted the first part of the simulations from the average calculation. Moreover, for selected homopeptides, we also repeated the pulling protocol in the opposite direction and repeated the calculation of pore clogging *b*^*eq*^, see Section S5 and Table S3. No significant differences are observed, suggesting that we sampled an equilibrium state and not a highly stretched conformation induced by the SMD. For reader convenience, we mention that protocols that allow to introduce solutes inside biological nanopores reducing possible conformational distortions induced by the pulling force were proposed in the literature^58,59^.

### Current measurement

We then select the frame for which the *Cα* of the central residue of the homopeptide is closer to the pore constriction defined as the average position of the seven copies of amino acid Met-113 of the *α*HL heptamer. This configuration was used for non-equilibrium runs where uniform and constant external electric field **E** = (0, 0, *E*_z_) was applied perpendicularly to the lipid bilayer. This protocol was shown to be equivalent to the application of a constant voltage Δ*V* = *E*_z_*L*_z_ ^38,60,61^. Each simulation was run for 240 ns and snapshots are saved every Δ*t* = 40 ps. The average current in the interval [*t*, *t* + Δ*t*] is estimated as

5 $$I(t)=\frac{1}{{\rm{\Delta }}t\,{L}_{{\rm{z}}}}\sum _{i=1}^{N}\,{q}_{{\rm{i}}}[{z}_{{\rm{i}}}(t+{\rm{\Delta }}t)-{z}_{i}(t)]$$

where *q*_i_ and *z*_i_ are charge and the z-coordinate of the *i*-th atom, respectively. The *K*^+^ and *Cl*^−^ currents were computed by restricting the sum over the atoms of corresponding type^38^. The mean current is obtained via a time average of *I*(*t*) after discarding a transient of 64 ns. Details on the statistical comparison of the current traces are reported in section S1 of the Supporting Information. As often occours in all-atom simulations, the driving voltage does not reflect the typical experimental conditions, but it is necessary for reducing the noise/signal ratio of the ionic current measurements. Current measurement have been carried out at Δ*V* = 1*V*. Although Δ*V* = 1*V* we can be outside of the linear response region, see e.g. extensive simulation at various voltages reported in^55,62^, we expect that the relative blockage Δ*I*/*I*_0_ is not strongly affected by the large Δ*V*.

## Supplementary information

Supporting information

## References

[1] Robertson, J. W. & Reiner, J. E. The utility of nanopore technology for protein and peptide sensing. *Proteomics* 1800026 (2018). 10.1002/pmic.201800026 PMC10935609 29952121

[2] Celaya G, Perales-Calvo J, Muga A, Moro F, Rodriguez-Larrea D (2017). Label-free, multiplexed, single-molecule analysis of protein–dna complexes with nanopores. ACS nano.

[3] Giamblanco, N. *et al*. Detection of protein aggregate morphology through single antifouling nanopore. *Sensors and Actuators B: Chemical* (2018).

[4] Waduge P (2017). Nanopore-based measurements of protein size, fluctuations, and conformational changes. ACS nano.

[5] Di Ventra M, Taniguchi M (2016). Decoding dna, rna and peptides with quantum tunnelling. Nat. nanotechnology.

[6] Huang G, Willems K, Soskine M, Wloka C, Maglia G (2017). Electro-osmotic capture and ionic discrimination of peptide and protein biomarkers with frac nanopores. Nat. communications.

[7] Gu L-Q, Cheley S, Bayley H (2003). Electroosmotic enhancement of the binding of a neutral molecule to a transmembrane pore. Proc. Natl. Acad. Sci..

[8] Kennedy E, Dong Z, Tennant C, Timp G (2016). Reading the primary structure of a protein with 0.07 nm^3^ resolution using a subnanometre-diameter pore. Nat. nanotechnology.

[9] Piguet F (2018). Identification of single amino acid differences in uniformly charged homopolymeric peptides with aerolysin nanopore. Nat. communications.

[10] Li, S., Cao, C., Yang, J. & Long, Y.-T. Detection of peptides with different charges and lengths by using the aerolysin nanopore. *Chem. Electro. Chem*.

[11] Chinappi M, Cecconi F (2018). Protein sequencing via nanopore based devices: a nanofluidics perspective. J. Physics: Condens. Matter.

[12] Diederichs, T., Nguyen, Q. H., Urban, M., Tampé, R. & Tornow, M. Transparent nanopore cavity arrays enable highly parallelized optical studies of single membrane proteins on chip. *Nano letters* (2018). 10.1021/acs.nanolett.8b01252 29741381

[13] Oukhaled A, Bacri L, Pastoriza-Gallego M, Betton J-M, Pelta J (2012). Sensing proteins through nanopores: fundamental to applications. ACS chemical biology.

[14] Restrepo-Pérez L, Joo C, Dekker C (2018). Paving the way to single-molecule protein sequencing. Nat. nanotechnology.

[15] Rossini AE, Gala F, Chinappi M, Zollo G (2018). Peptide bond detection via graphene nanogaps: a proof of principle study. Nanoscale.

[16] Heerema SJ (2018). Probing dna translocations with inplane current signals in a graphene nanoribbon with a nanopore. ACS nano.

[17] Zhao Y (2014). Single-molecule spectroscopy of amino acids and peptides by recognition tunnelling. Nat. nanotechnology.

[18] Rodriguez-Larrea D, Bayley H (2013). Multistep protein unfolding during nanopore translocation. Nat. nanotechnology.

[19] Nivala J, Marks DB, Akeson M (2013). Unfoldase-mediated protein translocation through an [alpha]-hemolysin nanopore. Nat. Biotechnol..

[20] Asandei A (2015). Placement of oppositely charged aminoacids at a polypeptide termini determines the voltage-controlled braking of polymer transport through nanometer-scale pores. Sci. reports.

[21] Tian K, Decker K, Aksimentiev A, Gu L-Q (2017). Interference-free detection of genetic biomarkers using synthetic dipole-facilitated nanopore dielectrophoresis. ACS nano.

[22] Chinappi M, Luchian T, Cecconi F (2015). Nanopore tweezers: Voltage-controlled trapping and releasing of analytes. Phys. Rev. E.

[23] Tian K, He Z, Wang Y, Chen S-J, Gu L-Q (2013). Designing a polycationic probe for simultaneous enrichment and detection of micrornas in a nanopore. ACS nano.

[24] Ciuca, A. *et al*. Single molecule, real-time dissecting of peptide nucleic acids-dna duplexes with a protein nanopore tweezer. *Anal. chemistry* (2018). 10.1021/acs.analchem.8b01568 29799733

[25] Asandei A (2016). Electroosmotic trap against the electrophoretic force near a protein nanopore reveals peptide dynamics during capture and translocation. ACS applied materials & interfaces.

[26] Boukhet M (2016). Probing driving forces in aerolysin and *α*-hemolysin biological nanopores: electrophoresis versus electroosmosis. Nanoscale.

[27] Di Marino D, Bonome EL, Tramontano A, Chinappi M (2015). All-atom molecular dynamics simulation of protein translocation through an *α*-hemolysin nanopore. The journal of physical chemistry letters.

[28] Bonome, E. L. *et al*. Multistep current signal in protein translocation through graphene nanopores. *The J. Phys. Chem. B* (2015). 10.1021/acs.jpcb.5b02172 25866995

[29] Ammenti A, Cecconi F, Marini Bettolo Marconi U, Vulpiani A (2009). A statistical model for translocation of structured polypeptide chains through nanopores. J. Phys. Chem. B-Condensed Phase.

[30] Szymczak P (2016). Periodic forces trigger knot untying during translocation of knotted proteins. Sci. reports.

[31] Cressiot, B., Oukhaled, A., Bacri, L. & Pelta, J. Focus on protein unfolding through nanopores. *Bio Nano Science*, 1–8 (2014).

[32] Oukhaled G (2007). Unfolding of proteins and long transient conformations detected by single nanopore recording. Phys. review letters.

[33] Wilson J, Sloman L, He Z, Aksimentiev A (2016). Graphene nanopores for protein sequencing. Adv. Funct. Mater..

[34] Luan B, Zhou R (2018). Single-file protein translocations through graphene–mos2 heterostructure nanopores. The journal of physical chemistry letters.

[35] Asandei A, Rossini AE, Chinappi M, Park Y, Luchian T (2017). Protein nanopore-based discrimination between selected neutral amino acids from polypeptides. Langmuir.

[36] Farimani, A. B., Heiranian, M. & Aluru, N. R. Identification of amino acids with sensitive nanoporous mos2: towards machine learning-based prediction. *Nat. 2D Mater*. **2** (2018).

[37] Song L (1996). Structure of staphylococcal alpha-hemolysin, a heptameric transmembrane pore. Sci..

[38] Aksimentiev A, Schulten K (2005). Imaging *α*-hemolysin with molecular dynamics: ionic conductance, osmotic permeability, and the electrostatic potential map. Biophys. journal.

[39] Isralewitz B, Gao M, Schulten K (2001). Steered molecular dynamics and mechanical functions of proteins. Curr. Opin. Struct. Biol..

[40] Zamyatnin A (1972). Protein volume in solution. Prog. biophysics molecular biology.

[41] Bhattacharya S, Yoo J, Aksimentiev A (2016). Water mediates recognition of dna sequence via ionic current blockade in a biological nanopore. ACS nano.

[42] Humphrey W (1996). Vmd: visual molecular dynamics. J. molecular graphics.

[43] Si W, Aksimentiev A (2017). Nanopore sensing of protein folding. ACS nano.

[44] Martin HS, Jha S, Howorka S, Coveney PV (2009). Determination of free energy profiles for the translocation of polynucleotides through *α*-hemolysin nanopores using non-equilibrium molecular dynamics simulations. J. chemical theory computation.

[45] Darby, N. J. & Creighton, T. E. *Protein structure* (Oxford University Press, USA, 1993).

[46] Bonella S, Raimondo D, Milanetti E, Tramontano A, Ciccotti G (2014). Mapping the hydropathy of amino acids based on their local solvation structure. The J. Phys. Chem. B.

[47] Miller S, Janin J, Lesk AM, Chothia C (1987). Interior and surface of monomeric proteins. J. molecular biology.

[48] Stoddart D (2014). Functional truncated membrane pores. Proc. Natl. Acad. Sci..

[49] Asandei Alina, Dragomir Isabela, Di Muccio Giovanni, Chinappi Mauro, Park Yoonkyung, Luchian Tudor (2018). Single-Molecule Dynamics and Discrimination between Hydrophilic and Hydrophobic Amino Acids in Peptides, through Controllable, Stepwise Translocation across Nanopores. Polymers.

[50] Phillips JC (2005). Scalable molecular dynamics with namd. J. computational chemistry.

[51] Brooks BR (2009). Charmm: the biomolecular simulation program. J. computational chemistry.

[52] Jorgensen WL, Chandrasekhar J, Madura JD, Impey RW, Klein ML (1983). Comparison of simple potential functions for simulating liquid water. The J. chemical physics.

[53] Luo Y, Roux B (2009). Simulation of osmotic pressure in concentrated aqueous salt solutions. The J. Phys. Chem. Lett..

[54] Comer, J. R., Wells, D. B. & Aksimentiev, A. Modeling nanopores for sequencing dna. In *DNA Nanotechnology*, 317–358 (Springer, 2011). 10.1007/978-1-61779-142-0_22 21674382

[55] Bonome EL, Cecconi F, Chinappi M (2017). Electroosmotic flow through an *α*-hemolysin nanopore. Microfluid. Nanofluidics.

[56] Lomize MA, Lomize AL, Pogozheva ID, Mosberg HI (2006). Opm: orientations of proteins in membranes database. Bioinforma..

[57] Thevenet P (2012). Pep-fold: an updated de novo structure prediction server for both linear and disulfide bonded cyclic peptides. Nucleic acids research.

[58] Wells DB, Abramkina V, Aksimentiev A (2007). Exploring transmembrane transport through *α*-hemolysin with grid-steered molecular dynamics. The J. chemical physics.

[59] Mathé J, Aksimentiev A, Nelson DR, Schulten K, Meller A (2005). Orientation discrimination of single-stranded dna inside the *α*-hemolysin membrane channel. Proc. Natl. Acad. Sci. United States Am..

[60] Crozier PS, Henderson D, Rowley RL, Busath DD (2001). Model channel ion currents in nacl-extended simple point charge water solution with applied-field molecular dynamics. Biophys. journal.

[61] Gumbart J, Khalili-Araghi F, Sotomayor M, Roux B (2012). Constant electric field simulations of the membrane potential illustrated with simple systems. Biochimica et Biophys. Acta (BBA)-Biomembranes.

[62] Bhattacharya S (2011). Rectification of the current in *α*-hemolysin pore depends on the cation type: the alkali series probed by molecular dynamics simulations and experiments. The J. Phys. Chem. C.

