# Supplementary material for: Insights into protein sequencing with an α-Hemolysin nanopore by atomistic simulations
Source: Sci Rep. 2019 Apr 23;9:6440. doi: 10.1038/s41598-019-42867-7 (PMC6478933; doi:10.1038/s41598-019-42867-7)
Supplement: Supplementary file 1 — Supporting information [file 41598_2019_42867_MOESM1_ESM.pdf]

Supporting information for "Insights into protein sequencing with an α-Hemolysin nanopore by atomistic simulations"

Giovanni Di Muccio[1,+], Aldo Eugenio Rossini[2,+], Daniele Di Marino[3,4,*], Giuseppe Zollo[2], and Mauro Chinappi[1,]**

[1]Dipartimento di Ingegneria Industriale, Università di Roma Tor Vergata, Via del Politecnico 1, 00133, Roma, Italia
[2]Dipartimento di Scienze di Base e Applicate per l'Ingegneria (Sezione di Fisica), Università di Roma "La Sapienza", Via A. Scarpa 14–16, 00161 Rome, Italy
[3]Faculty of Biomedical Sciences, Institute of Computational Science - Center for Computational Medicine in Cardiology Università della Svizzera Italiana (USI)
[4]Polytechnic University of Marche, Department of Life and Environmental Sciences, Via Brecce Bianche, 60131 Ancona, Italy.
[*]daniele.di.marino@usi.ch
[**]mauro.chinappi@uniroma2.it
[+]these authors contributed equally to this work

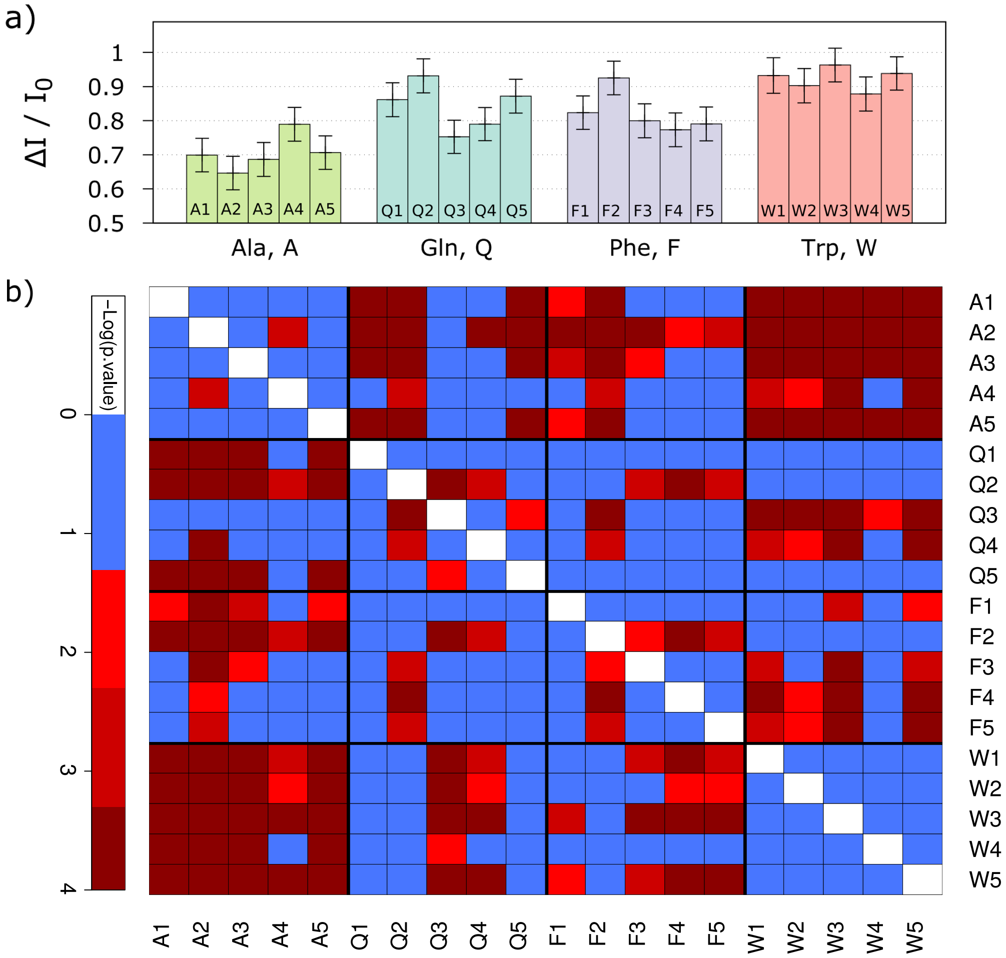

Figure S1. a) Average current blockage $\Delta I/I_0 = (I_0 - I)/I_0$, with I the average current measured with the homopeptide inside the pore and I_0 the empty pore value, for each single replica of the four homopeptides Ala, Phe, Gln, Trp. Significant differences among replicas for the same homopeptide are found for Ala, Phe and Gln. b) Student's t-test adjusted p-value matrix. Each row (and column) refers to a single replica, where, following the same nomenclature of panel a, the letter corresponds to the amino acid forming the homopeptide and the number refers to the replica. Red squares represent significant differences, adjusted $p < 0.05$, while light blue ones refer to non significant differences (adjusted $p > 0.05$).

S1. Statistical analysis of ionic currents

For each one of the four homopeptides, we sampled 5 independent series of data, for a total of 20 ionic current tracks. The average blockades are shown in Fig. S1a. Each series is composed by $N = 4400$ uncorrelated current data points corresponding to the average current value over 40 ps intervals. We verified that each the time series is uncorrelated, using different methods as: i) block average approach as reported in Allen and Tildesley[1], ii) the calculation of effective sample size using *coda* package in R[2,3]. To evaluate if the average ionic current I measured for one replica is significantly different from another one, a series of Student's t-test (null hypothesis: the average current is the same) has been conducted. Fig. S1b reports the p-value matrix of t-test between each couple of samples. Multiple test correction is performed using the Holm-Bonferroni strategy[4]. Red boxes indicates $p < 0.05$, while light blue ones refer to $p > 0.05$. All tests are computed using R[3].

For completeness, we would like to cite that noise in a single current trace is usually due to both thermal fluctuations and to changes in molecule conformations inside the pore. The latter dynamics is usually slower than thermal fluctuations and can introduce a gradual variation of the blockade current on relatively long time scales, the interested reader can see the data and the analysis reported in[5]. Our simulations are not long enough to explore large conformational changes within a single run and to apply the proposed in[5]. In our work, possible conformational differences are taken into account by repeating the simulation for five different replicas for each peptide and estimating the error bars (figure 1 of the manuscript) considering current blockades from independent replicas as independent measurements.

S2. Determination of the $A(z)$ profile

Occupancy maps have been calculated using Volmap VMD plugin[6]. The system is divided in cubic cells of size $\Delta x = \Delta y = \Delta z = 1$Å, and, for each frame, we used the VMD Volmap plug-in[6] to compute the occupancy map of the electrolyte, $m_{\mathrm{x,y,z}}$, where x, y, z indicate the cell, $m_{\mathrm{x,y,z}} = 1$ if the center of the cell is within a Van der Waals radius of at least one water or ion atoms and $m_{\mathrm{x,y,z}} = 0$ elsewhere. Then, we averaged $m_{\mathrm{x,y,z}}$ over all frames and normalized it with the bulk value, obtaining an averaged and normalized occupancy map indicated with $M_{\mathrm{x,y,z}}$. The map $M_{\mathrm{x,y,z}}$ for the case of empty αHL at equilibrium is reported in Fig. S2a. Reentrant pockets, early discussed in[7], are apparent, see, e.g. red arrow S2a. In addition, $M_{\mathrm{x,y,z}}$ is different from zero also outside the pore. To select only the area inside the pore and to exclude the reentrant pockets, we implemented the following procedure sketched in Fig. S2e. As a first step, we selected a threshold M_0. Then, we choose a single cell at the trans pore mouth for which $M_{\mathrm{x,y,z}} > M_0$, this is the first cell of the trans→cis channel, see Fig. S2b,II. Starting from this cell we searched whatever or not neighbouring cells on the same z-section or in the trans→cis direction (left, right and top in a 2D representation of Fig. S2b,II, highlighted with a yellow dashed line) have occupancy $M_{\mathrm{x,y,z}} > M_0$. If $M_{\mathrm{x,y,z}} > M_0$, the cell is added to the list of trans→cis channel cells. The procedure is then repeated on the just added trans→cis cells and so on, each step being reported in panels from II to VIII of S2b. This procedure defines the trans→cis channel and exclude the reentrant pockets, see the sketch in S2b-IX. The same approach is carried out starting from the cis mouth leading to a cis→trans channel, Fig. S2b,X. Then, final channel is obtained as the intersection between the cis→trans and trans→cis channels, i.e. only cells belonging to both of them, are considered, see fig S2b,XI. This final map, is the one indicated as $\tilde{M}_{\mathrm{x,y,z}}$ in the paper and used for the estimation of the accessible section $A(z)$. Figure S2a-d report the original map (a), the trans→cis channel (b), the cis→trans channel (c) and final channels (d) for the case of empty αHL.

S3. Comparison between non-equilibrium and equilibrium pore clogging estimators

Table S1 reports the non-equilibrium current blockage estimator b and equilibrium one b^{eq} for Ala, Phe, Gln and Trp averaged over five replicas. For Ala, Gln and Tpr, both indicators have the same ranking, i.e. the lower value of the pore clogging estimator corresponds to Ala, the larger to Trp and the intermediate to Gln, i.e. the same ranking observed in the average measured currents (Fig. 1b of the paper).

However, equilibrium estimators b^{eq} are, in general, slightly larger than the non-equilibrium ones b. Such a behaviour is associated with another observation: at equilibrium, the main and secondary constrictions have a slightly smaller radius compared to the non-equilibrium system, as shown in Fig. S3a where the inverse accessible area profiles $A(z)^{-1}$ for empty αHL are reported for equilibrium (purple solid) and non-equilibrium (dashed green). In the following, we report a simplified model that explains the connection between these two observations. Let us consider a cylindrical pore of length L and section A, see Fig. S3b. The electrical resistance R is given by

$$R = \frac{\rho L}{A} \,, \tag{1}$$

with ρ resistivity of the electrolyte. The pore clogging estimator is hence,

$$b = 1 - \frac{R_0}{R} = 1 - \frac{A_{\mathrm{c}}}{A_0} \,, \tag{2}$$

where R_0 and A_0 are the electric resistance and electrolyte accessible area for the empty pore, R is the resistance of the clogged pore and A_{c} is the area available for the electrolyte passage when the peptide is into the pore, see Fig. S3b. As a first approximation, we can model the peptide chain as a cylinder of area A_{aa}. Assuming that A_{aa} is the same for both equilibrium and non-equilibrium runs, the electrolyte accessible area for the clogged pore are expressed as

$$A_{\mathrm{c}}^{eq} = A_0^{eq} - A_{\mathrm{aa}} \quad , \quad A_{\mathrm{c}}^{neq} = A_0^{neq} - A_{\mathrm{aa}} \tag{3}$$

and, hence, from equation (2), we get

$$b^{eq} = \frac{A_{\mathrm{aa}}}{A_0^{eq}} \quad , \quad b = \frac{A_{\mathrm{aa}}}{A_0^{neq}} \,. \tag{4}$$

As anticipated, our simulations indicates that for empty pore the accessible area is larger for non-equilibrium runs, Fig S3a. Recast in the present simplified model, this means $A_0^{eq} < A_0^{neq}$ and, consequently,

$$b^{eq} > b \,. \tag{5}$$

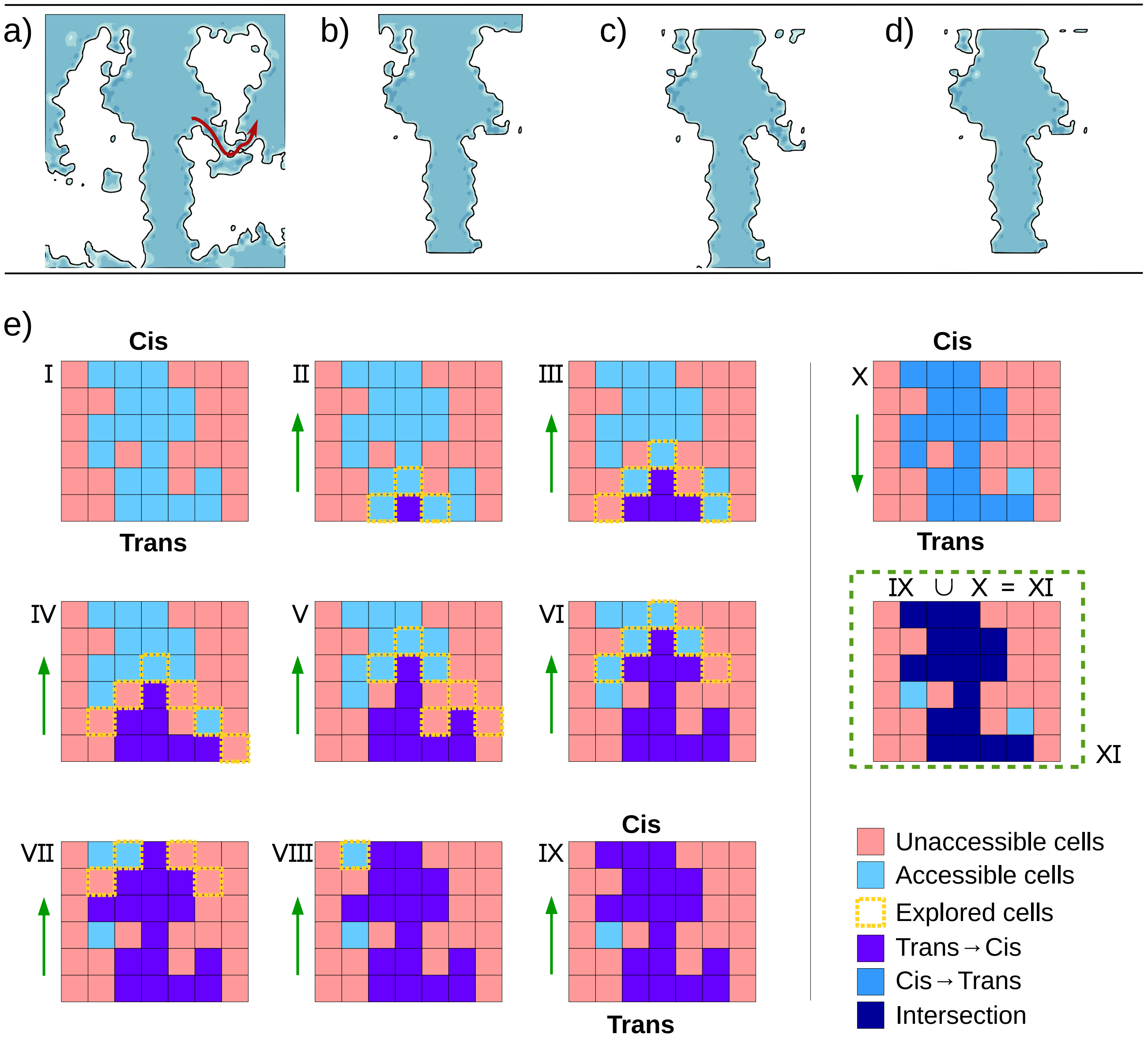

Figure S2. Accessible volume. a) Averaged and normalized map $M_{x,y,z}$. Blue areas correspond to region that are fully accessible by the electrolyte $M_{x,y,z} = 1$ while white ones correspond to $M_{x,y,z} = 0$. Water reentrant pockets, already observed in[7] are apparent close to the constriction (red arrow in panel a). Although these pockets are connected to the electrolyte region inside the pore, they do not contribute to the electrolyte motion between the two sides of the membrane. To filter our these dead-end pockets, we set up an iterative described in section S2 and sketched in panel (e), that allowed us to define a trans→cis and an cis→trans maps, panels (b) and (c). The intersection between these two maps leads to the final map $\tilde{M}_{x,y,z}$, panel (d).

Homopeptide	b	b^{eq}
Ala	0.16 ± 0.006	0.16 ± 0.007
Gln	0.22 ± 0.015	0.27 ± 0.013
Phe	0.29 ± 0.011	0.38 ± 0.021
Trp	0.36 ± 0.019	0.42 ± 0.032

Table S1. Non-equilibrium, b, and equilibrum, b^{eq}, pore clogging estimators for the Ala, Gln, Trp and Phe homopeptides. The data are obtained averaging on 5 indipendent replica for each homopeptide.

Moreover, $b^{eq} - b$ reads

$$b^{eq} - b = A_{\mathrm{aa}} \left(\frac{1}{A_0^{eq}} - \frac{1}{A_0} \right) , \qquad (6)$$

i,e, it increases with the amino acid size. This is in qualitative agreement with the Ala, Gln and Trp data, indeed, for Ala, $b^{eq} \simeq b$ while for the other cases, $b^{eq} > b$.

For Phe, instead, the Phe equilibrium clogging estimator is much larger that the non-equilibrium one. This is probably due due to a large clogging peak in the replica F1, as shown in Fig. S3c. For comparison, we reported the inverse of the accessible area also for Tyrosine (Fig. S3d) an amino acid of volume similar to Phe.

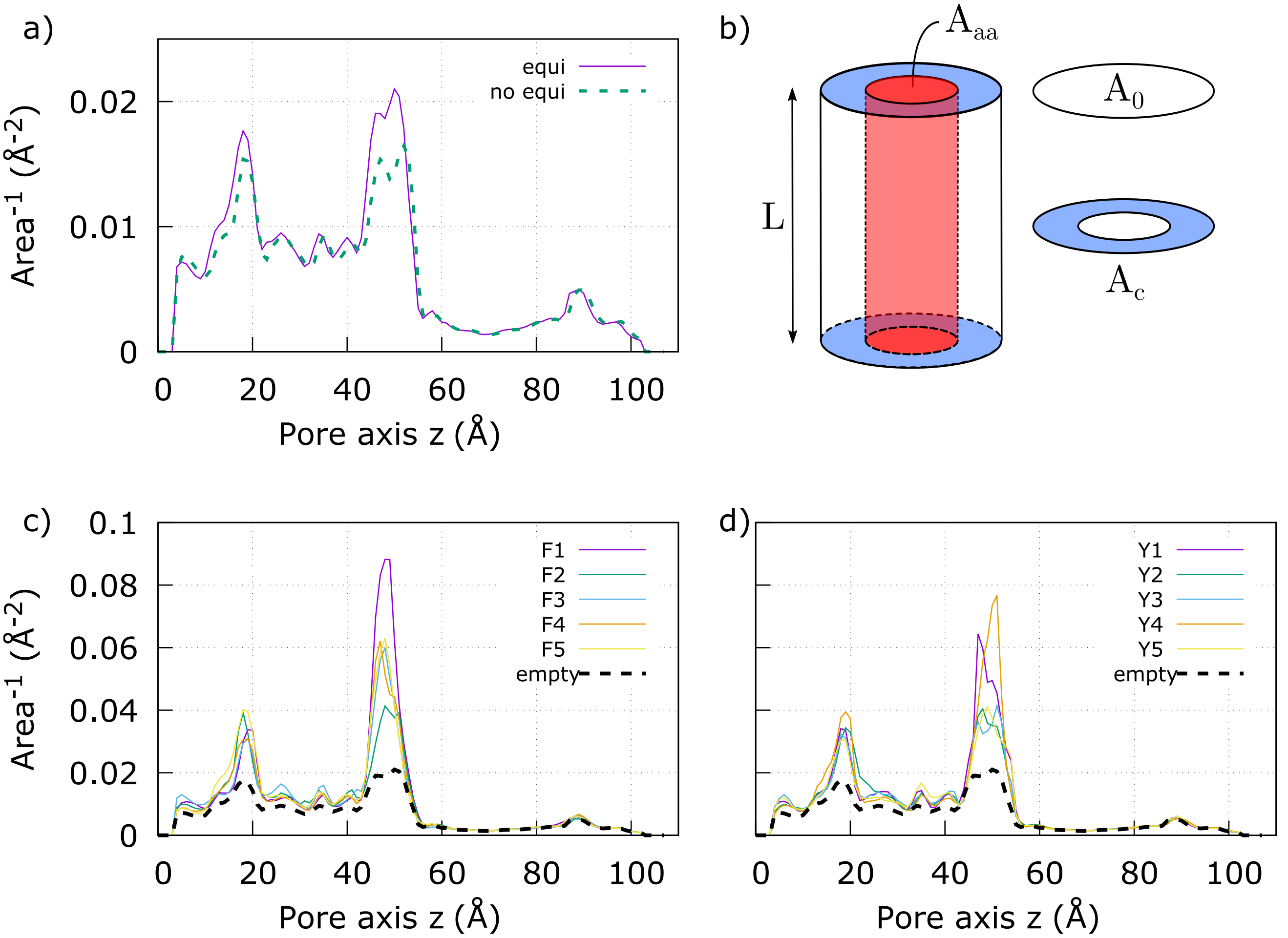

Figure S3. a) Inverse of the accessible area, A_z for the empty pore in the equilibrium simulation run (purple solid line), compared to the non-equilibrium one (green dashed line). It can be noted that the equilibrium sampling estimates a larger blockage of the two constrictions, indicating an effect of the electric field on those areas. b) Sketch of the cylindrical model discussed in section S3. A_0 is the section of the empty cylinder, A_{aa} the radius of the clogging chain and $A_c = A_0 - A_{aa}$ the available section for the electrolyte. c-d) Inverse of the accessible area, A_z , along the pore for Phenilalanine (c, F) and Tyrosine (d, Y) homopetides for equilibrium runs.

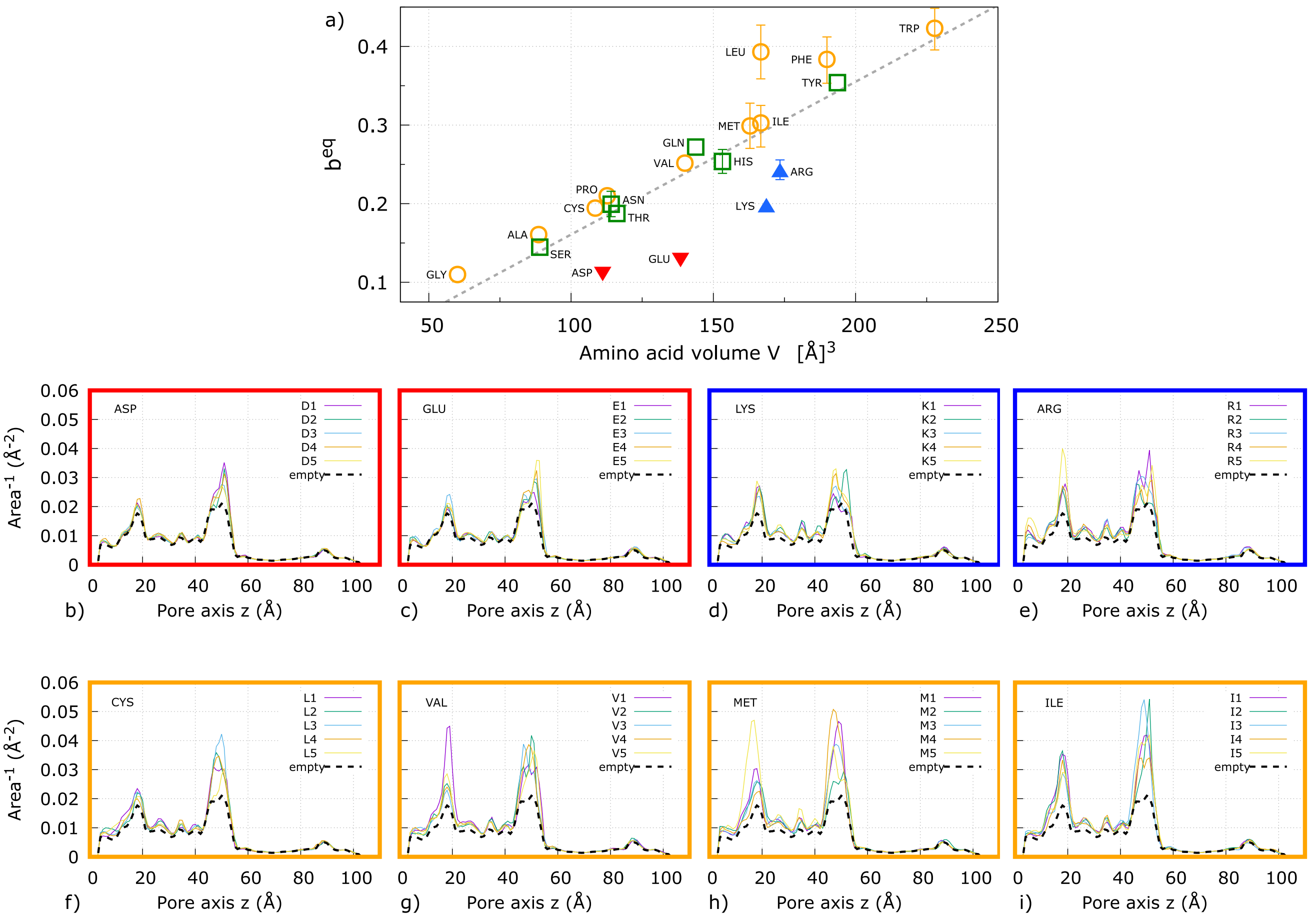

Figure S4. Comparison between charged and hydrophobic amino acids. Panel (a) reports the same data of Fig. 4a of the paper, i.e pore clogging estimator b^{eq} for all residues Vs the amino acid volume V_a. Yellow circles corresponds to hydrophobic residues, green squares to polar ones, blue up-triangles to positively charged residues and red down-triangles to negatively charged ones. The dashed line is the minimum square fit. Error bars are estimated by considering b^{eq} from independent replicas as independent measurements and they are reported only when larger than symbols. Panels (b-e) reports the inverse of the accessible area, A_z, along the pore for charged (red and blue) amino acids, while panels (f)-(i) refer to the non-polar (yellow) homopetides the volume of which is the closest one to the corresponding charged residue, e.g. Cys for Asp, Val for Glu, Met for Lys and Ile for Arg. For comparison, in each plot, also the empty pore profile (dashed line) is reported.

S4. Comparison between b^{eq} for hydrophobic and polar residues

To quantify the difference between b^{eq} for hydrophobic and polar residues, we defined for each amino acid the quantity

$$\Delta b^{eq} = b^{eq} - r(V_{\mathrm{aa}}) \,, \tag{7}$$

where b^{eq} is the pore clogging equilibrium estimator and $r(V_{\mathrm{aa}})$ the clogging estimator value obtained from the minimum square fit using the volume V_{aa} of the *aa* amino acid. In essence, Δb^{eq} is the vertical distance (with sign) from a data point and the minimum square line in Fig. 4a of the paper. We then performed a t-test between the group constituted by hydrophobic amino acids and the group formed by polar (not charged) ones. The p-value of the test is $p = 0.054$.

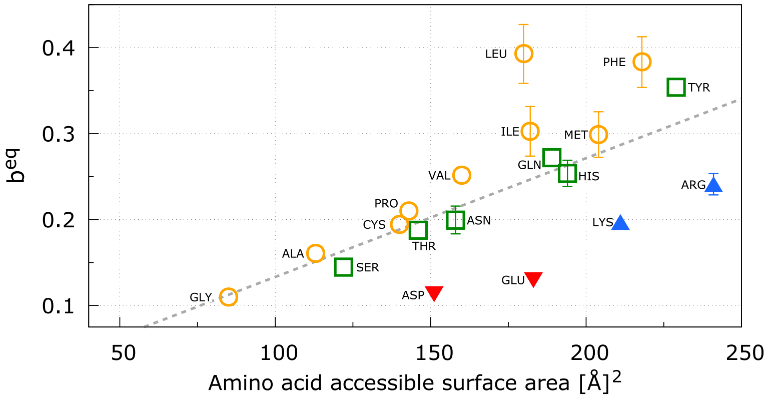

Figure S5. Pore clogging estimator b^{eq} for all residues Vs the amino acid accessible surface area S as defined in[8]. Yellow circles corresponds to hydrophobic residues, green squares to polar ones, blue up-triangles to positively charged residues and red down-triangles to negatively charged ones. The dashed line is the minimum square fit. Error bars are estimated by considering b^{eq} from independent replicas as independent measurements and they are reported only when larger than symbols.

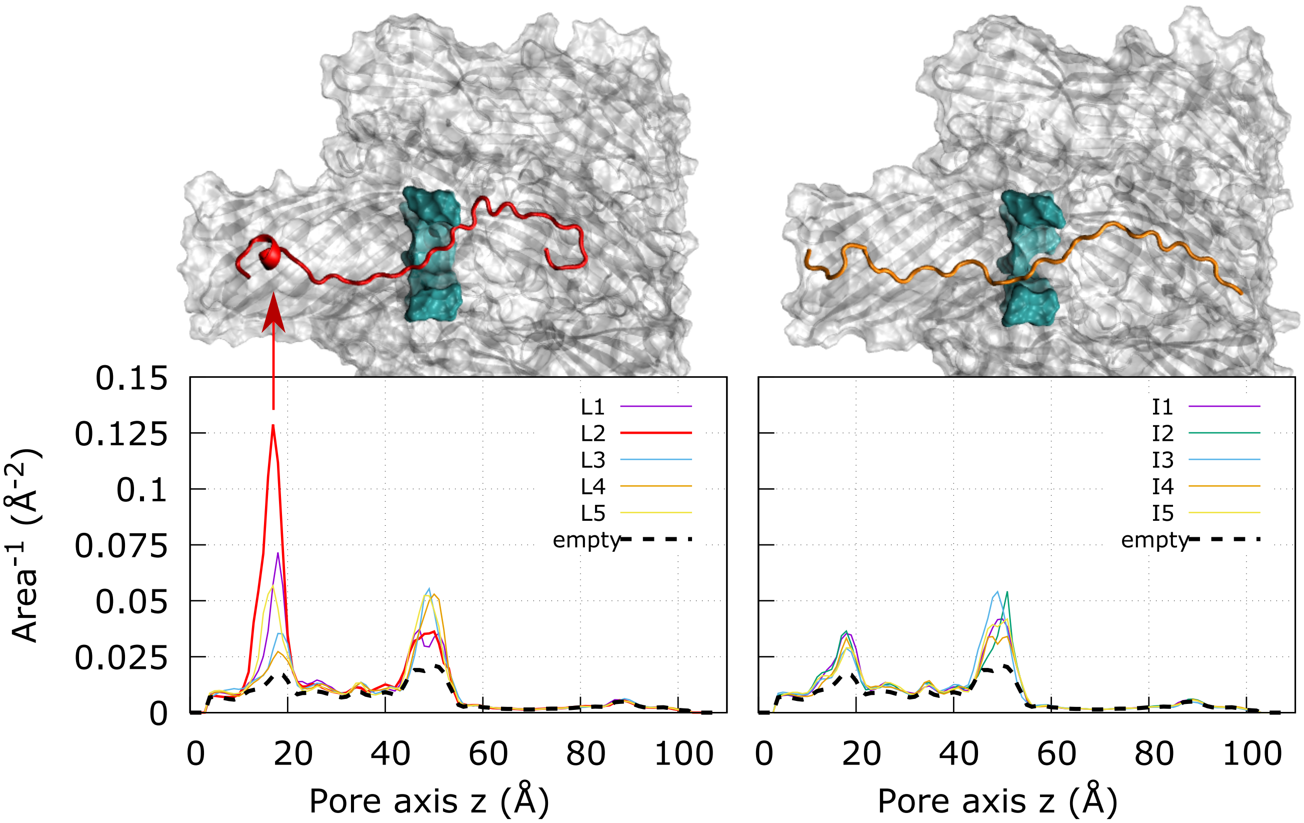

Figure S6. Inverse of the accessible area, A_z , along the pore for Leucine (left, L) and Isoleucine (right, I) homopetides. The clogging of the primary central constriction, $z \simeq 50$Å, is very similar for the two isomers while the secondary barrel constriction, $z \simeq 20$Å, shows a higher clogging for the Leucine replicas L1, L2 and L5. These peaks are due to the presence of short helices in the barrel region. In the replica L2, the highest peak the α-helix persists for the entire run, a snapshot is reported on the top left. In L1 and L5, the helix folds and unfolds several times during the run suggesting that this conformation is metastable. For isoleucine case, instead, no secondary structures form during the runs.

S5. Structural analysis of charged and unchaged peptides

Both charged and uncharged homopeptides inside the transmembrane region of αHl are, for all cases, in a quite linear conformation. To characterize the stretching state of the homopeptide in the pore narrowest region (considered here from Met-113 to Leu-135), we calculated the average of the distance along the pore axis between two consecutive C_α, i.e. $d_z = |z_{\mathrm{i}}^{C\alpha} - z_{\mathrm{i+1}}^{C\alpha}|$ where $z_{\mathrm{i}}^{C\alpha}$ is the z-coordinate of the C_α of the $i-th$ peptide amino acid. The average distance $\langle d_z \rangle$ for equilibrium run, calculated skipping the first the 32 ns (where the homopeptide is still relaxing, see section S6 and Fig. S7), is slightly larger for the charged peptides with respect to uncharged ones. No differences are observed among different groups of uncharged residues, see table S2. This indicates that actually the charged peptides are more stretched leaving more room to the electrolyte as apparent in their reduced pore clogging index b^{eq} with respect to uncharged peptides of similar volume. Interestingly, counting the water molecules and the ions atoms inside the barrel, it results that the biggest difference between the charged and uncharged peptides is the water/ions ratio. For instance, for Lys the average water number inside the barrel is the same of Ile, even if Lys presents in average one less residue inside the barrel with respect to Ile; the ions instead are 70% more for Lys than for Ile. Similar results are obtained with the other charged residues.

Amino acid type	Homopeptides	$\langle d_z \rangle$
Charged	Asp, Glu, Lys, Arg	3.035 ± 0.041
Uncharged	All non charged	2.761 ± 0.030
Polar	Ser, Thr, Asn, Gln, His, Tyr	2.779 ± 0.043
Non-polar	Gly, Ala, Cys, Pro, Val, Ile, Leu, Met, Phe	2.749 ± 0.042

Table S2. Average values of axial distance between C_α of consecutive amino acids, $\langle d_z \rangle$, for different group of amino acids.

S6. Peptide Relaxation

To avoid to include in the calculation of b^{eq} also data corresponding to highly stretched conformations, we monitored the time evolution of the gyration radius of the polymers during the production runs. Fig. S7 reports 4 representative cases corresponding to the simulations with $\mathbf{E} = 0$ used to calculate pore clogging estimator b_{eq}. Exponential fits indicate that the typical relaxation time is $\simeq$ 10 ns and that, in none of the case, it is larger than 20 ns. For this reason, we selected to skip the first 32 ns in the calculation of b^{eq}. In addition, we repeated our protocol pulling the homopeptide in the opposite direction (i.e. from Cis to Trans during the SMD run) for selected homopeptides (Ala, Trp, Gln). In this way, possible preferential alignments of the lateral chain due to the pulling direction should be the opposite than the ones corresponding to our original pulling strategy. The data for b^{eq}, reported in Tab. S3, show that there are no significant differences with original data where homopeptides are imported from Trans to Cis, further supporting that the homopeptide reach a conformation that, to some extent, loses memory of the pulling strategy.

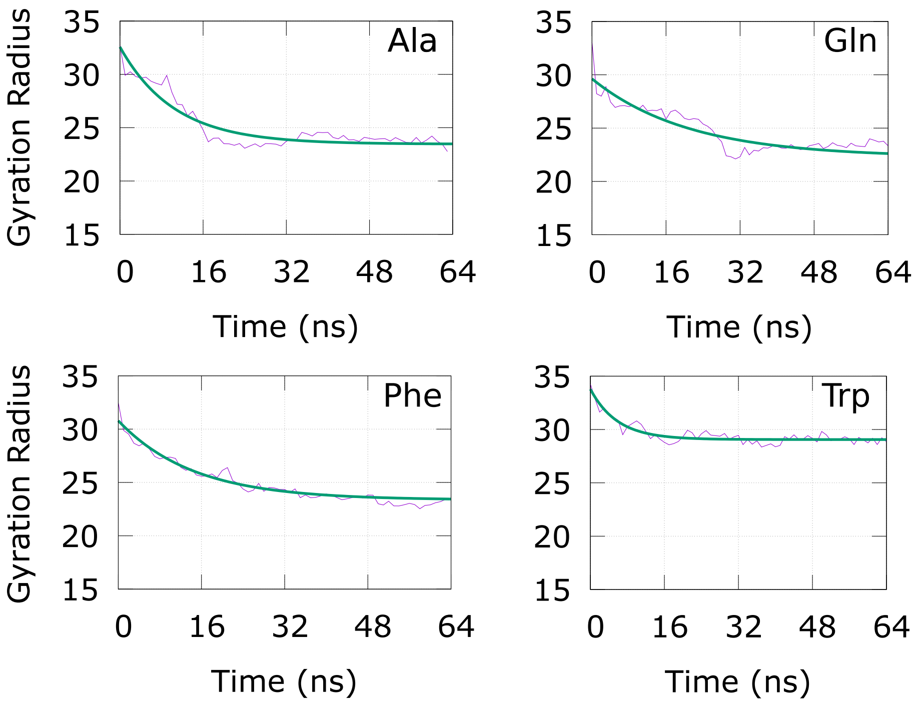

Figure S7. Gyration radius of four homopeptides (violet). The gyration radius relaxes in few decades of nanoseconds. To estimate the characteristic decay time τ, the curves are fitted with an exponential function (green), $R_{\mathrm{g}}(t) = A\exp(-t/\tau) + B$. The average τ for the four homopeptides is 10.6 ns.

Homopeptide	b^{eq} $SMD_{\mathrm{Cis}\rightarrow\mathrm{Trans}}$	b^{eq} $SMD_{\mathrm{Trans}\rightarrow\mathrm{Cis}}$
Ala	0.16 ± 0.015	0.16 ± 0.006
Gln	0.26 ± 0.015	0.27 ± 0.012
Trp	0.40 ± 0.028	0.42 ± 0.032

Table S3. Equilibrum b^{eq} pore clogging estimators for Ala, Gln and Trp homopeptides, computed on systems initialized by opposite SMD insertion protocols, Trans $\rightarrow$ Cis versus Cis $\rightarrow$ Trans, see section S6. The values are obtained averaging on 5 independent replicas for the $\mathrm{SMD}_{\mathrm{Trans}\rightarrow\mathrm{Cis}}$ systems, and on 3 independent replicas for the $\mathrm{SMD}_{\mathrm{Cis}\rightarrow\mathrm{Trans}}$ systems.

References

1. Allen, M. P. & Tildesley, D. J. *Computer simulation of liquids* (Oxford university press, 2017).
2. Plummer, M., Best, N., Cowles, K. & Vines, K. Coda: Convergence diagnosis and output analysis for mcmc. *R News* **6**, 7–11 (2006).
3. R Core Team. *R: A Language and Environment for Statistical Computing*. R Foundation for Statistical Computing, Vienna, Austria (2013).
4. Holm, S. A simple sequentially rejective multiple test procedure. *Scand. journal statistics* 65–70 (1979).
5. Bhattacharya, S., Yoo, J. & Aksimentiev, A. Water mediates recognition of dna sequence via ionic current blockade in a biological nanopore. *ACS nano* **10**, 4644–4651 (2016).
6. Humphrey, W., Dalke, A., Schulten, K. *et al.* Vmd: visual molecular dynamics. *J. molecular graphics* **14**, 33–38 (1996).
7. Aksimentiev, A. & Schulten, K. Imaging α-hemolysin with molecular dynamics: ionic conductance, osmotic permeability, and the electrostatic potential map. *Biophys. journal* **88**, 3745–3761 (2005).
8. Miller, S., Janin, J., Lesk, A. M. & Chothia, C. Interior and surface of monomeric proteins. *J. molecular biology* **196**, 641–656 (1987).
